# Investigation into the use of a chitosan scaffold for tarsal plate substitution in eyelid reconstruction

**DOI:** 10.1093/rb/rbaf114

**Published:** 2025-11-07

**Authors:** Glyn Chidlow, John P M Wood, Luke A Halliday, Robert J Casson, Shaveen S B Gamage, Andrea J O’Connor, Dinesh Selva, Michelle T Sun

**Affiliations:** Discipline of Ophthalmology & Visual Sciences, Level 7 Adelaide Health and Medical Sciences Building, University of Adelaide, North Terrace, Adelaide, SA 5000, Australia; South Australian Institute of Ophthalmology, Royal Adelaide Hospital, Adelaide, SA 5000, Australia; Discipline of Ophthalmology & Visual Sciences, Level 7 Adelaide Health and Medical Sciences Building, University of Adelaide, North Terrace, Adelaide, SA 5000, Australia; South Australian Institute of Ophthalmology, Royal Adelaide Hospital, Adelaide, SA 5000, Australia; Discipline of Ophthalmology & Visual Sciences, Level 7 Adelaide Health and Medical Sciences Building, University of Adelaide, North Terrace, Adelaide, SA 5000, Australia; South Australian Institute of Ophthalmology, Royal Adelaide Hospital, Adelaide, SA 5000, Australia; Discipline of Ophthalmology & Visual Sciences, Level 7 Adelaide Health and Medical Sciences Building, University of Adelaide, North Terrace, Adelaide, SA 5000, Australia; South Australian Institute of Ophthalmology, Royal Adelaide Hospital, Adelaide, SA 5000, Australia; Department of Biomedical Engineering, Graeme Clark Institute, The University of Melbourne, Melbourne, VIC 3010, Australia; Department of Biomedical Engineering, Graeme Clark Institute, The University of Melbourne, Melbourne, VIC 3010, Australia; Discipline of Ophthalmology & Visual Sciences, Level 7 Adelaide Health and Medical Sciences Building, University of Adelaide, North Terrace, Adelaide, SA 5000, Australia; South Australian Institute of Ophthalmology, Royal Adelaide Hospital, Adelaide, SA 5000, Australia; Discipline of Ophthalmology & Visual Sciences, Level 7 Adelaide Health and Medical Sciences Building, University of Adelaide, North Terrace, Adelaide, SA 5000, Australia; South Australian Institute of Ophthalmology, Royal Adelaide Hospital, Adelaide, SA 5000, Australia

**Keywords:** tarsus, chitosan, hydrogel, scaffold, eyelid

## Abstract

We previously described the production of hydrophilic hydrogel scaffolds of cross-linked chitosan that closely matched the biomechanical properties of native human tarsus. In the present preclinical study we appraised the spatio-temporal tissue response to the implantation of these chitosan scaffolds into rat eyelids. Acellular hydrogel scaffolds were produced from Good Manufacturing Practice (GMP)-compliant chitosan and implanted into rat eyelids. Tissue was harvested and fixed up to 24 weeks post-surgery for histological examination of the tissue response to both the surgical procedure itself and to the chitosan implantation. Assessment encompassed inflammation, the foreign body reaction (FBR) and infiltration of native cells into the implant region, along with scaffold degradation. Three days after implantation of the chitosan into rat eyelids, neutrophils were observed in the vicinity of the chitosan but their prevalence declined rapidly thereafter. Both pro-inflammatory M1-macrophages and anti-inflammatory M2-macrophages were also observed post-implantation at the scaffold-tissue interface but the former cells declined after 4 weeks. Unlike the M1-macrophages, the M2-macrophages rarely infiltrated the scaffold at any time point. T cells and MHC class II antigen-presenting cells were predominantly increased at the tissue-scaffold interface and, to a minor degree, within the scaffold, in the weeks following surgery. In the weeks following implantation, a fibro-collagenous capsule gradually formed at the margins of the scaffolds, denoting the classic FBR. This was accompanied by the appearance of foreign body giant cells, moderate to substantial degradation and engulfment of chitosan by infiltrating cells, and localized tissue remodelling characterized by proliferation of fibroblasts, deposition of collagenous extracellular matrix (ECM) material and rudimentary formation of vascular elements within the scaffold. Although the chitosan scaffolds initially elicited widespread inflammation and an FBR, longer-term tissue remodelling and scaffold degradation suggested their biocompatibility. These data support that chitosan hydrogel scaffolds could, therefore, serve as suitable tarsal substitute material in situ.

## Graphical abstract

**Figure:**
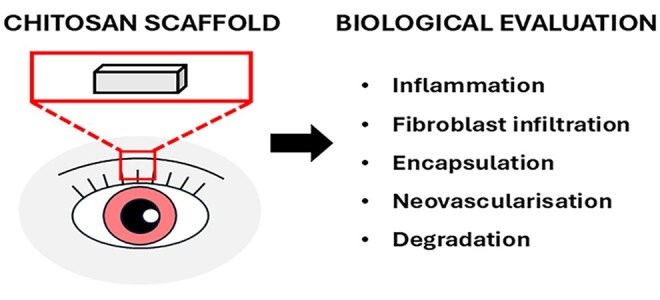

## Introduction

The eyelid, which plays a key role in protecting the eye, is divided into the anterior lamella, comprising skin and orbicularis oculi muscle, and the posterior lamella, encompassing the palpebral conjunctiva and the tarsus [1]. The tarsus consists of a region of dense, curved fibrocartilaginous connective tissue which provides structural support and incorporates the oil-secreting meibomian glands [2, 3]. Poor eyelid function can result in significant local morbidity and associated visual impairment [4]. For full thickness or deep posterior lamellar defects, successful surgical reconstruction requires an adequate tarsus substitute to maintain post-operative tissue structure [5]. Eyelid tarsus surrogates can be harvested either from autologous tissue or from xenogenic grafts but these are limited by an array of factors, including availability of substitute tissue, donor site morbidity, difficulty in replicating tarsal function and potential rejection [5]. Recently, bioengineering principles are being applied to develop novel tarsus tissue substitutes which have the potential to improve reconstructive outcomes by better replicating form and function [5].

Tissue engineering is based on a multidisciplinary approach to the repair or regeneration of complex tissues [6–8], which commonly involves producing a three-dimensional scaffold from a biocompatible polymeric material and implanting this into the desired surgical site to instigate autologous self-repair [9]. The scaffold can be cellular, i.e. pre-populated with appropriate cells that have been propagated *in vitro*, or acellular, whereby non-cellularized implants are populated *in situ* by infiltrating native cells [10]. After implantation, the scaffold should integrate and gradually degrade, ideally at a rate which parallels appropriate cellular infiltration. Eventually only functional tissue derived from incorporated, differentiated cells should remain [10]. Although several reports have described excellent candidate engineered synthetic scaffold material for tarsal substitution [11–20], there has been no progress in directly applying findings to the human tarsus, so their potential long-term merit cannot be appropriately assessed at present.

Scaffolds can be prepared from non-synthetic, natural polymers. Scaffolds derived from the natural microbial polymer, poly(3-hydroxybutyrate-co-3-hydroxyhexanoate) (PHBHHx), for example, have shown excellent promise in tarsal engineering [18]. Our laboratory is interested in exploring the potential of chitosan for tarsal bioengineering, given its promise in skin, bone cartilage, vascular tissue, dental and corneal tissue engineering [21–24]. Chitosan is a biopolymer that is prepared via alkaline-deacetylation of the abundant crustacean exoskeletal polysaccharide, chitin [25]. This polymer represents an excellent candidate for tarsal engineering because it is derived from an abundant natural source, possesses relatively low immunogenicity, forms water-swollen hydrogels, has antibacterial properties, and has a net cationic charge which promotes cell adhesion, proliferation and differentiation [23, 26]. Chitosan also has structural similarity to glycosaminoglycans which are present in the extracellular matrix (ECM) [2] and is therefore readily degraded by endogenous enzymes such as lysozyme *in situ* [27, 28].

Following implantation of a biomaterial into a tissue, a foreign body reaction (FBR) is initiated by the host [29, 30]. The FBR comprises a number of sequential steps deriving from blood/tissue-implant interaction, including blood protein adsorption onto the biomaterial surface, fibrin matrix formation, acute and chronic inflammation, and fusion of recruited macrophages to form foreign body giant cells [29, 30]. These giant cells aim to protect the host tissue by instigating both degradation of the foreign material and its encapsulation by fibroblast-derived collagen matrices [31]. Obviously, however, encapsulation of the implant is not ideal because this isolates it from the host, interfering with its integration and, thus, impairing its intended purpose. Thus, implantation of a material which engenders a more minimal FBR is obviously desirable.

We previously described the preparation and characterization of hydrophilic hydrogel scaffolds of cross-linked chitosan, that, importantly, closely matched the biomechanical properties determined for native human tarsus and were also able to support incorporation and growth of ocular fibroblasts [32]. In the present study, we produced these hydrogel scaffolds from GMP-compliant chitosan and implanted them into rat eyelids. We subsequently carried out a detailed examination of the post-operative tissue over a period of 24 weeks, to assess whether this material could serve as a suitable tarsal substitute *in situ*. We therefore investigated tissue inflammatory responses, and integration of the scaffolds, as indexed by infiltration of native cells. We also closely examined aspects of the FBR, including whether there was evidence for either encapsulation or scaffold degradation *in situ* during the time course of the study. Resultant data allowed us to assess the biocompatibility of the scaffold material, and, hence, its potential for use as a tarsal substitute in human patients in the future.

## Materials and methods

### Chitosan hydrogel scaffolds

Preparation of chitosan hydrogel scaffolds was as previously described, with minor modifications [32]. Good Manufacturing Practice (GMP)-compliant quality chitosan (Heppe Medical Chitosan GmbH, Germany; Chitoceuticals^®^ 80/500 GMP-compliant grade) was used in this study as an important step towards potential clinical application as changes in a biomaterial’s source and quality can significantly impact its biological interactions. Briefly, chitosan (5% w/v; degree of deacetylation 77.6–82.5%) was dissolved in 0.2 M acetic acid at 37°C for 24 h. The chitosan was then cross-linked by treating with freshly prepared 0.1% v/v glutaraldehyde and the solution subsequently degassed by centrifugation (4000 g, 4 min, room temperature). The resulting chitosan solution was introduced into rectangular glass moulds, sealed in plastic bags, and cooled in an aqueous bath of 50% (v/v) ethylene glycol at −20°C for 24 h for cryogelation, before being neutralized overnight in excess 3 M NaOH under orbital shaking at 120 rpm. Scaffolds were washed in water (3 × 3 min) and phosphate-buffered saline (PBS; 1 h, orbital shaking 180 rpm), followed by excess 0.1 M glycine in PBS (4 h, orbital shaking, 180 rpm) to remove or cap any unreacted glutaraldehyde. Scaffolds were disinfected by soaking in 80% (v/v) ethanol overnight and then stored in sterile PBS at 4°C until use. All solutions were made with ultrapure water purified to a resistivity of 18.2 MΩ. The biomechanical properties of the chitosan hydrogel were tailored to replicate those of human tarsal tissue including strain-stiffening and the ability to be stretched to 30% strain repeatedly without failure [32]. The scaffolds had a mean initial modulus, final modulus and extensibility of 0.113 ± 0.016 MPa, 0.455 ± 0.099 MPa and 17.2 ± 1.0% under tensile testing, respectively [32].

### Animals

This study was approved by the Animal Ethics Committee of The University of Adelaide (Adelaide, SA, Australia; approval number #33228) and conformed with the Australian Code of Practice for the Care and Use of Animals for Scientific Purposes, 2013, and with the Association for Research in Vision and Ophthalmology (ARVO) Statement for the use of animals in vision and ophthalmic research. A rat model was selected for this preclinical study as this is an established species for medical research and which has an eyelid morphology which is structurally similar to that of a human, meaning it provides good translational value, but with attendant logistical experimental advantages.

**Figure 1.**
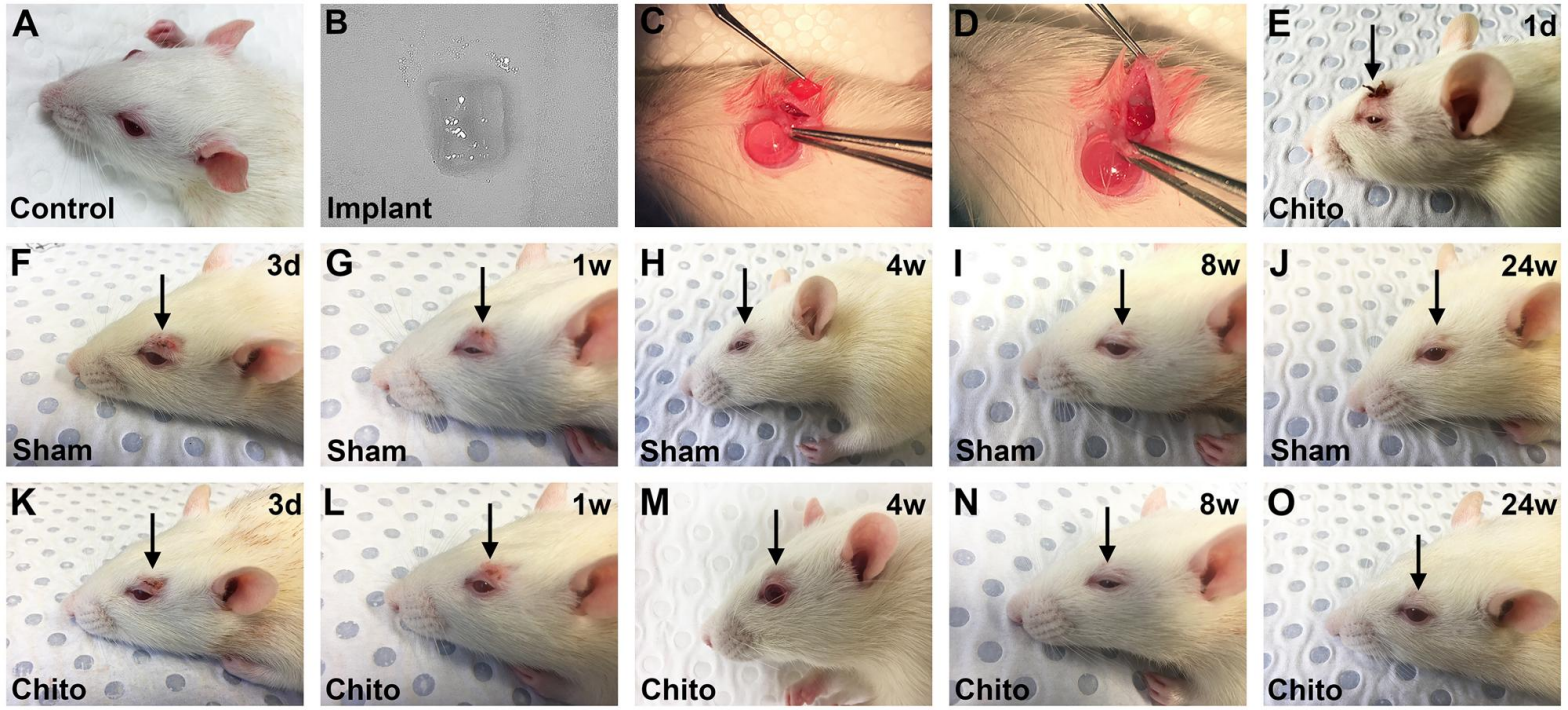
Surgical procedure and post-operative appearance of rat eyelids. (**A**) Appearance of an untreated adult Sprague-Dawley rat, showing normal eyelid. (**B**) Chitosan scaffold immediately prior to implantation, with transparent, gelatinous appearance. (**C**) Surgical incision made in the upper eyelid of the rat. (**D**) Implantation of chitosan scaffold into surgical site. (**E**) Clinical appearance at 1 day post-surgery after removal of dressing (chitosan animal shown as example; sham animal looked the same). Clinical appearance of sham-treated animals at (**F**) 3 days, (**G**) 1 week, (**H**) 4 weeks, (**I**) 8 weeks, (**J**) 24 weeks after surgery. Clinical appearance of chitosan-implanted animals at (**K**) 3 days, (**L**) 1 week, (**M**) 4 weeks, (**N**) 8 weeks, (**O**) 24 weeks after surgery. Note that, in both groups, the external scarring had essentially resolved by 4 weeks and the fur had fully grown back by 8 weeks. Arrows indicate site of surgery in each case.

Male Sprague-Dawley rats (*n* = 32), aged 10–12 weeks and weighing 300–350 g, were purchased from The University of Adelaide. Animals were housed in a humidity and temperature-controlled room with a 12-hour light and 12-hour dark cycle and provided with food and water *ad libitum*. Animals were randomly allocated into one of three treatment groups: chitosan implant (*n* = 18), sham surgery (*n* = 10) or untreated control (*n* = 4). Control rats received no surgery (Figure 1A). The rationale for using a sham group as well as an untreated control group is to isolate scaffold-specific tissue responses at each time point from those that relate purely to the surgical intervention. The untreated group represents the healthy eyelid against which the chitosan implant and sham surgery groups can be evaluated. Prior to surgery, rats were anaesthetized with an intraperitoneal injection of a mixture of 100 mg/kg ketamine and 10 mg/kg xylazine. The fur of the left upper eyelid was shaved to reveal the skin and local anaesthesia (0.2% bupivacaine with 5 μg/mL adrenaline) was then administered subcutaneously above the left orbital rim. After 15 min, a 5 mm skin incision was made in the left eyelid approximately 1 mm superior to the eyelid margin (Figure 1). Rats in the chitosan group received a 1.0 mm × 1.0 mm scaffold implant, while sham animals received no implant (Figure 1). All defects were closed with biodegradable sutures for the first three days post-surgery. Post-operatively, topical antibiotic ointment was applied to the wounds and a small dressing placed over the surgical sites for 1 day. Rats were monitored daily, and clinical photographs were taken at regular intervals coinciding with the analytical time-points. On a predetermined date after surgery, rats were humanely killed by transcardial perfusion with 0.9% w/v normal saline under terminal anaesthesia. Following euthanasia, the eyelids were dissected and fixed in 10% neutral buffered formalin for 24 h. After fixation, eyelids were processed for routine paraffin-embedding and oriented on-edge so that transverse sections could be prepared. The number of eyes analysed per treatment group at each time point was as follows: 3 days (chitosan, *n* = 3; sham, *n* = 2); 1 week (chitosan, *n* = 3; sham, *n* = 2); 4 weeks (chitosan, *n* = 3; sham, *n* = 2); 8 weeks (chitosan, *n* = 3; sham, *n* = 2); 24 weeks (chitosan, *n* = 6; sham, *n* = 2); A further group of untouched eyes (*n* = 4) served as controls, as mentioned previously.

### Histological stains

Tissue sections were stained for haematoxylin and eosin (H&E) using a standard methodology. For Masson’s trichrome stain for collagen, deparaffinized and rehydrated sections were stained with 0.5% (w/v) Celestin Blue/haematoxylin. This was followed with 1% (w/v) Biebrich Scarlet-1% (w/v) Acid Fuschin, in 1% (v/v) glacial acetic acid to stain all tissue components red. Subsequent differentiation with 5% (w/v) phosphotungstic acid removed the red colour only from collagenous ECM components. The final step was treatment with 2.5% (w/v) Aniline Blue, with differentiation in 1% (v/v) acetic acid. This stains collagen fibres dark blue in colour. Mast cells were identified by staining of rehydrated sections with 0.1% (w/v) toluidine blue (pH 2.3) for 3 min. Toluidine blue stains mast cell granules red-purple (metachromatic staining) with the background tissue stained blue (orthochromatic staining). Details for all histological stains were as previously reported [33].

### Immunohistochemistry

Colorimetric immunohistochemistry was performed as previously described [33, 34]. In brief, tissue sections were deparaffinized and treated with 0.5% (v/v) H_2_O_2_ to block endogenous peroxidase activity. Antigen retrieval was achieved by heating the sections in a microwave oven immersed in 10 mM citrate buffer (pH 6.0) for 10 min at 95–100°C. For localization of collagens IV and V and laminin, sections received an additional digestion for 10 min with proteinase K (20 µg/mL) to unmask antigen sites. Subsequently, sections were incubated in primary antibody (see Table 1 for details), followed by consecutive incubations with biotinylated secondary antibody and streptavidin-peroxidase conjugate. Colour development was achieved using 3,3’-diaminobenzidine and images of labelled sections were captured using a standard light microscope (BX51; Olympus, Mount Waverly, Victoria, Australia) with attached vibration-free camera.

**Table 1.** Antibodies

Antibody	Host	Dilution	Company	Catalogue
α-SMA	Mouse	1:2000	Dako	M0851
CD206	Rabbit	1:50,000	Abcam	ab64693
CD3	Rabbit	1:2500	Dako	A0452
Collagen IV	Rabbit	1:1000	Abcam	ab6586
Collagen V	Goat	1:1000	Chemicon	ab781
ED1	Mouse	1:500	Bio-Rad	MCA341R
eNOS	Mouse	1:1000	BD Transduction	N30020
Iba1	Goat	1:5000	Novus Biologicals	NB100-1028
iNOS	Rabbit	1:500	BD Transduction	610332
Laminin	Rabbit	1:2500	EY Labs	AT 2404
MHC class II	Goat	1:2000	Santa Cruz	sc-5438
Myeloperoxidase	Rabbit	1:50,000	Dako	A 0398
Vimentin	Mouse	1:5000	Dako	M0725

For double-labelling fluorescent immunohistochemistry, visualization of one antigen was achieved using a three-step procedure (primary antibody, biotinylated secondary antibody, streptavidin-conjugated AlexaFluor 488 or 594), while the second antigen was concurrently labelled by a two-step procedure (primary antibody, secondary antibody conjugated to AlexaFluor 488 or 594). Sections were prepared as above, then incubated overnight at room temperature in the appropriate combination of primary antibodies. On the following day, sections were incubated with the appropriate biotinylated secondary antibody for the three-step procedure plus the correct secondary antibody conjugated to AlexaFluor 488 or 594 for the two-step procedure, followed by streptavidin-conjugated AlexaFluor 488 or 594. Sections were then mounted using anti-fade mounting medium and examined under standard microscope with epifluorescence optics (BX-61; Olympus) equipped with a scientific grade, cooled charge-coupled device (CCD) camera.

Confirmation of the specificity of antibody labelling was judged by the morphology and distribution of the labelled cells, by the absence of signal when the primary antibody was replaced by isotype/serum controls, and by comparison with the expected staining pattern in based on our own, and other, previously published results. The relative abundances of various cells in three regions, namely, within the scaffold, at the tissue-scaffold boundary (peri-scaffold), and > 0.5 mm from the scaffold, were assessed by two independent, blinded observers and visually scored on a semi-quantitative scale from − (not detectable) to +++ (high prevalence). Scaffold degradation was evaluated using the following semi-quantitative scale: − (no scaffold degradation), + (minor cracks/fragments), ++ (multiple cracks/fragments), +++ (pronounced fragmentation/degradation).

## Results

Post-operatively, rats regained consciousness within one hour and returned to normal eating habits and behaviour thereafter. Wound healing was uncomplicated in all treatment groups, scab formation and resolution occurred in the first week, growth of hair occurred from approximately two to four weeks, and full external healing had occurred by eight weeks in all groups (Figure 1). The operated eyelids were functionally normal when compared to the unoperated eye, and the cornea and lens remained clear and healthy in all treatment groups at all time points (Figure 1).

### Tissue responses to chitosan implant

The objective of the present study was to provide spatio-temporal data pertaining to inflammatory, immune and wound healing responses to implantation of a chitosan scaffold into rat eyelids subjected to a tarsal plate defect. To achieve this aim, a combination of histological and immunohistochemical evaluations were performed at 3 days, 1 week, 4 weeks, 8 weeks and 24 weeks after surgery. For each parameter, evaluations were made in three regions of the eyelid, namely, within the scaffold, at the tissue-scaffold boundary (peri-scaffold), and >0.5 mm from the scaffold using semi-quantitative grading. Untreated control animals provided the baseline for each parameter of interest. Importantly, comparisons were also made against sham-operated rats at each time point in order to differentiate between scaffold-specific tissue responses versus those that relate purely to the surgical procedure.

### Acute inflammation

**Figure 2.**
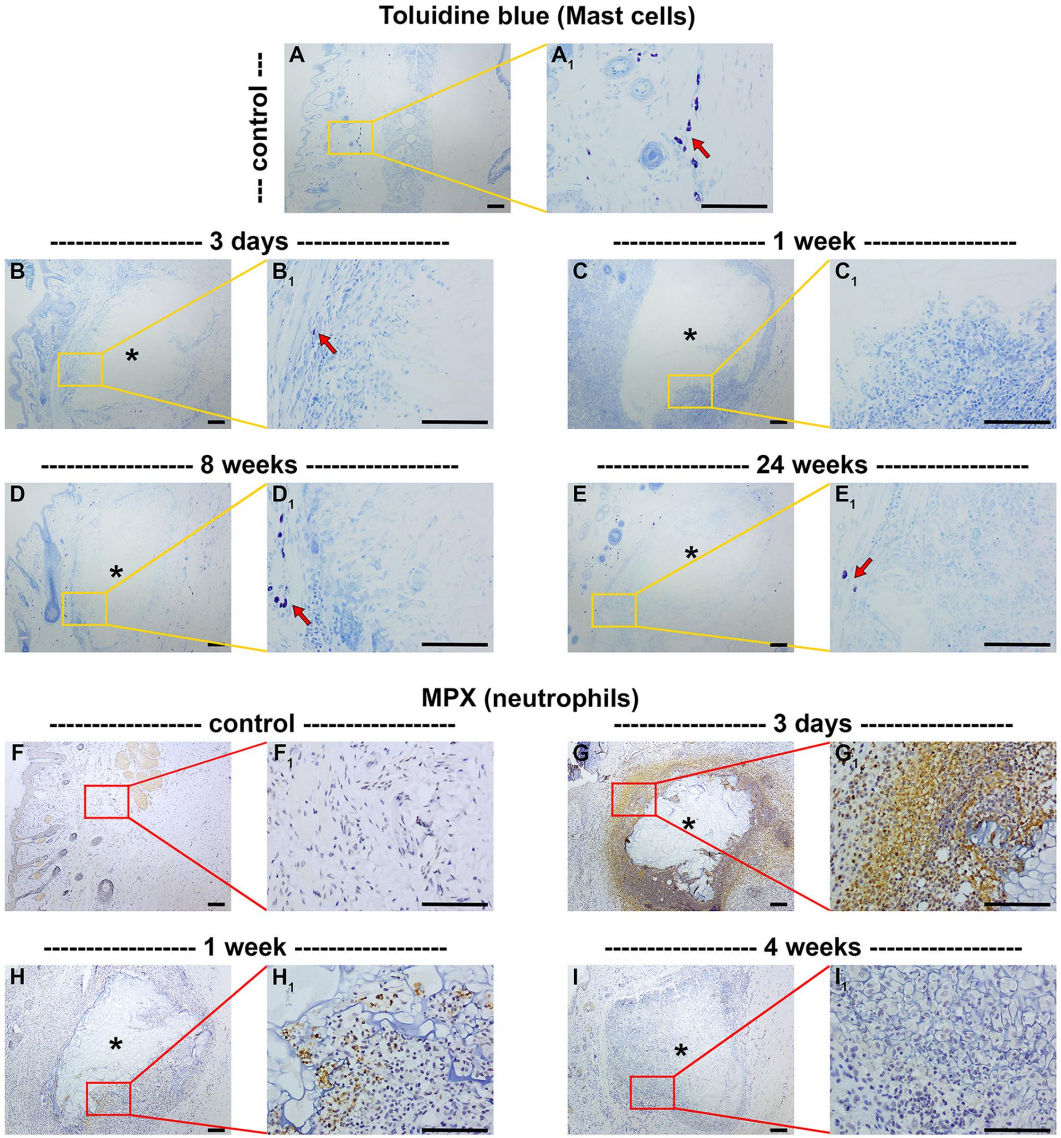
Representative images of mast cells (**A–E**) and neutrophils (**F–I**) in paraffin-embedded sections of rat eyelids implanted with chitosan scaffolds, as delineated by toluidine blue staining and colorimetric immunohistochemistry for myeloperoxidase (MPX), respectively. At each time point, low (**A–I**) and corresponding high (**A_1_–I_1_**) magnification images are shown. (**A**) In normal eyelid tissue, mast cells (arrow) are only infrequently observed. (**B–E**) Following implant surgery, the abundance of mast cells within or surrounding the scaffold did not increase, irrespective of time point analysed. (**F**) In normal eyelid tissue, MPX^+^ neutrophils are not observed. (**G–I**) At 3 days following surgery, there were many MPX^+^ cells surrounding and invading the scaffold. By one week after surgery, there were fewer MPX^+^ cells at the scaffold site, and by 4 weeks after surgery, MPX^+^ cells were scarce. Asterisks represent scaffolds. Scale bars: 100 µm.

The acute inflammatory tissue response to implanted biomaterials is characterized by infiltration of polymorphonuclear granulocytes, principally neutrophils and mast cells. In healthy rat eyelids, mast cells and neutrophils were scarce and not detectable, respectively. Following sham surgery or implantation of chitosan scaffold, the number of mast cells within, surrounding, or distal to, the scaffold did not increase, at any of the time points analysed (Figure 2, Table 2). In contrast, neutrophils were abundant at the chitosan scaffold at 3 days following implant surgery, but their prevalence rapidly declined thereafter (Figure 2, Table 2). In sham-operated rats, there was a very modest, transient increase in neutrophils (Figure 2, Table 2).

**Table 2.** Semi-quantitative labelling grading

Target	Location	3 days	1 week	4 weeks	8 weeks	24 weeks
Toluidine blue (mast cells)	Scaffold	–	–	–	–	–
	Peri-scaffold	−/+	−/+	−/+	−/+	−/+
	>0.5 mm from scaffold	+	+	+	+	+
	Sham surgery	+	+	+	+	+
MPX (neutrophils)	Scaffold	+/++	+/++	+	−/+	−/+
	Peri-scaffold	+++	+/++	−/+	−/+	−/+
	>0.5 mm from scaffold	+/++	−/+	–	–	–
	Sham surgery	+	−/+	–	–	–
ED1 (macrophages)	Scaffold	+/++	+/++	++	++/+++	+++
	Peri-scaffold	+++	+++	+/++	−/+	−/+
	>0.5 mm from scaffold	++	++	+	–	–
	Sham surgery	++	+/++	−/+	–	–
iNOS (M1 macrophages)	Scaffold	+/++	+/++	++	+/++	++/+++
	Peri-scaffold	++	+/++	−/+	−/+	−/+
	>0.5 mm from scaffold	–	–	–	–	–
	Sham surgery	–	–	–	–	–
CD206 (imDCs and M2 macrophages)	Scaffold	–	–	+/++	+/++	−/+
	Peri-scaffold	+	+/++	+/++	+/++	++
	>0.5 mm from scaffold	++/+++	++/+++	++	+/++	+/++
	Sham surgery	++/+++	++	+/++	+	+
CD74 (MHC II)	Scaffold	–	−/+	+	+/++	+/++
	Peri-scaffold	++	++	++	++	++
	>0.5 mm from scaffold	++/+++	++	+	+	+
	Sham surgery	++	++	−/+	−/+	−/+
CD3 (T cells)	Scaffold	–	−/+	+	+	+
	Peri-scaffold	+	++	++	++	++
	>0.5 mm from scaffold	++	+/++	+	−/+	−/+
	Sham surgery	+/++	+/++	+	−/+	−/+
Vimentin (mesenchymal cells)	Scaffold	+/++	+/++	++	++/+++	+++
	Peri-scaffold	++	+++	++	++	++
	>0.5 mm from scaffold	++	++	++	+/++	+/++
	Sham surgery	++	++	+	+	+
Collagenous ECM	Scaffold	–	−/+	+	+/++	++/+++
	Peri-scaffold (capsule)	+	++	++	++	++
Basement membrane ECM scaffold degradation	Scaffold	–	–	−/+	+	+/++
	Scaffold	–	–	+	++	++/+++

### Chronic inflammation

**Figure 3.**
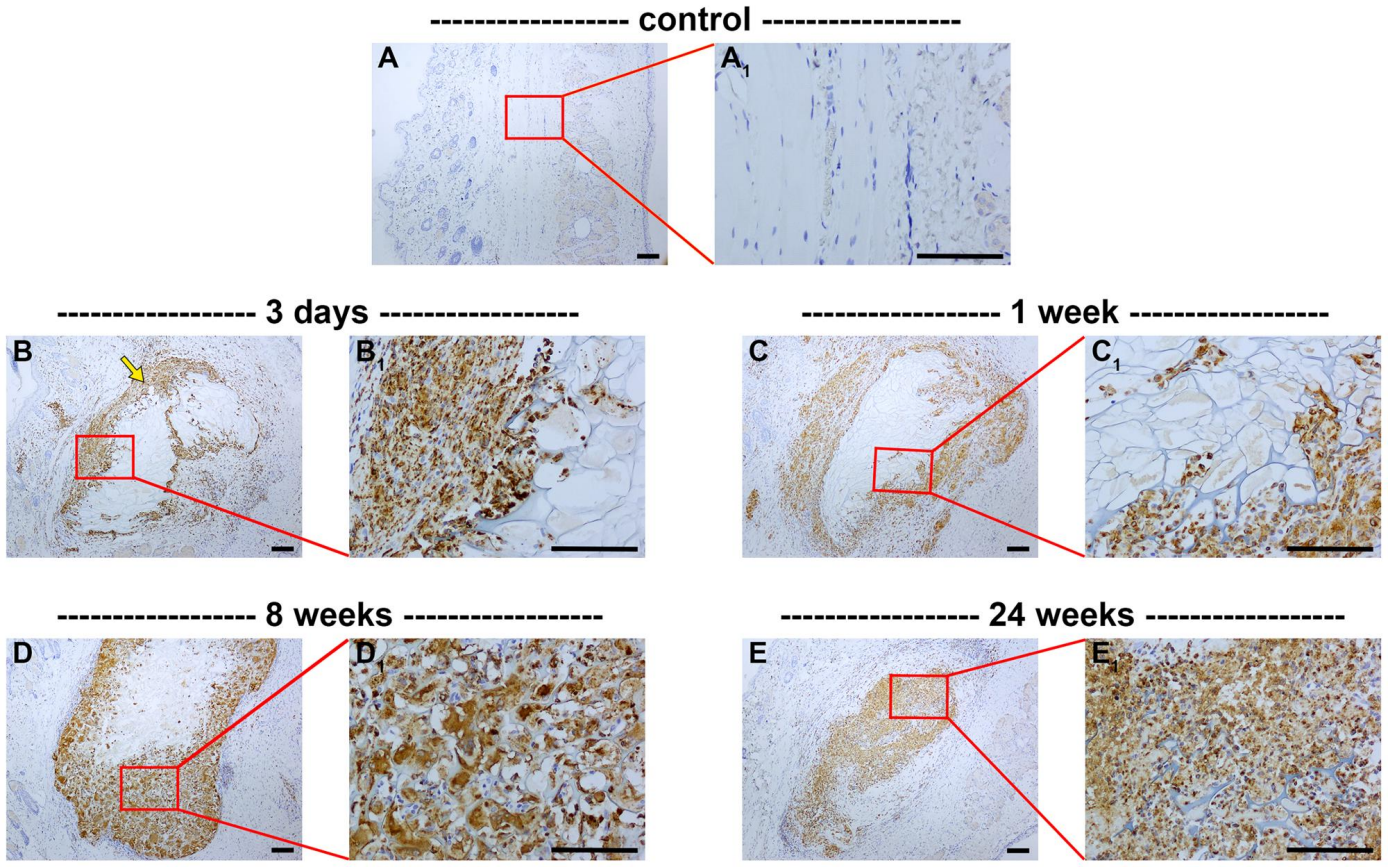
Representative images of monocytes/macrophages in paraffin-embedded sections of rat eyelids implanted with chitosan scaffolds, as delineated by colorimetric immunohistochemistry for the pan-macrophage marker ED1. At each time point, low (**A–E**) and corresponding high (**A_1_–E_1_**) magnification images are shown. (**A**) In normal eyelid tissue, ED1^+^ cells are not observed. (**B–E**) Following surgery, ED1^+^ cells were observed surrounding and invading the scaffold at all time points investigated. The abundance of ED1^+^ cells within the scaffold increased throughout the 24-week period of observation. Scale bars: 100 μm.

The chronic inflammatory response to implanted biomaterials typically comprises infiltration of monocytes and lymphocytes. Monocytes subsequently differentiate into macrophages, which are considered the predominant cells in tissue responses to implanted biomaterials [30]. ED1, an antibody that binds to the rat CD68 antigen, demarcates monocytes and all classes of macrophages [35]. In normal eyelid tissue, ED1^+^ cells were not observed (Figure 3A). Following surgery, however, ED1^+^ cells were observed surrounding and infiltrating the scaffold (Figure 3B). The abundance of ED1^+^ cells within the scaffold gradually increased throughout the 24-week period of observation (Figure 3B–E). In contrast, few ED1^+^ cells were observed outside of the implant after 4 weeks (Figure 3D and E, Table 2). In sham-operated rats, there was a modest increase in ED1^+^ cells for the first week after surgery (Table 2).

**Figure 4.**
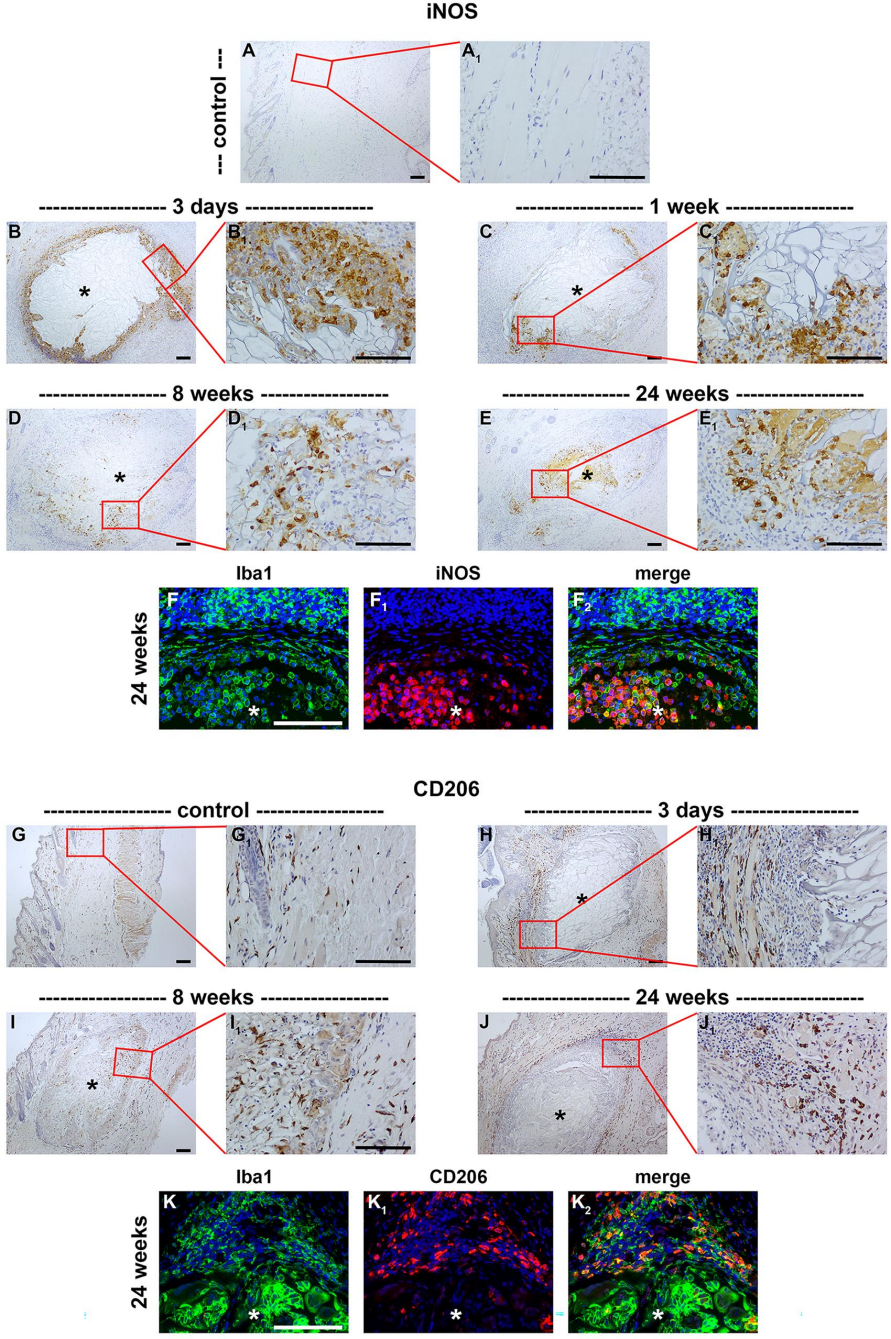
Representative images of putative M1 and M2 macrophages in paraffin-embedded sections of rat eyelids implanted with chitosan scaffolds, as delineated by colorimetric immunohistochemistry for iNOS (**A–F**) and CD206 (**G–K**), respectively. At each time point, low (**A–J**) and corresponding high (**A_1_–J_1_**) magnification images are shown. (**A**) In normal eyelid tissue, iNOS^+^ cells are not observed. (**B–E**) At 3 days following implant surgery, many iNOS^+^ cells were observed surrounding the scaffold. The abundance of iNOS^+^ cells in the peri-scaffold region progressively decreased at 1, 8 and 24 weeks after surgery. Conversely, the amount of iNOS^+^ cells within the scaffold increased over the same time period. (**F**) Double labelling of iNOS with the macrophage/dendritic cell marker Iba1 at 24 weeks reveals that iNOS co-localized with Iba1 in many cells with the morphology of macrophages within the implant. (**G**) In normal eyelid tissue, CD206^+^ cells are scattered throughout the eyelid. (**H–J**) At 3 days following implant surgery, the number of CD206^+^ cells increased throughout the eyelid and surrounding the scaffold. By 8 weeks, CD206^+^ cells were also observed within the scaffold. By 24 weeks, CD206^+^ cells were largely absent from the scaffold itself and concentrated in the peri-scaffold region. (**K**) Double labelling of CD206 with the macrophage/dendritic cell marker Iba1 at 24 weeks reveals that CD206 co-localized with Iba1 in many cells surrounding, but not within, the implant. Asterisks represent scaffolds. Scale bars: 100 µm.

Macrophages represent a heterogeneous population of cells. They have been classified into pro-inflammatory, phagocytic M1-type macrophages, or anti-inflammatory, pro-regenerative M2-type macrophages. To shed light on macrophage phenotypes associated with implanted chitosan scaffolds, we employed stereotypical markers of M1 and M2 macrophages, namely iNOS and CD206, respectively. In normal eyelid tissue, iNOS^+^ cells were not observed, but 3 days following surgery iNOS^+^ cells surrounded the implant (Figure 4, Table 2). The abundance of iNOS^+^ cells in the peri-scaffold region decreased over time, whereas the amount of iNOS^+^ cells within the scaffold increased (Figure 4, Table 2). In sham-operated rats, iNOS^+^ cells were likewise not observed (Table 2). CD206 is a validated marker of M2 macrophages. Additionally, it is expressed by immature dendritic cells. In control tissue, CD206^+^ dendritic cells were scattered throughout the eyelid (Figure 4). In the first week after surgery, the number of CD206^+^ cells increased throughout the eyelid (Figure 4, Table 2). At the scaffold-tissue interface, CD206^+^ cells with the morphology of macrophages were observed at all time points analysed (Figure 4, Table 2). In contrast, few CD206^+^ cells were evident within the scaffold, other than a modest presence at 4–8 weeks (Figure 4, Table 2). In sham-operated rats, CD206^+^ cells, were abundant for the first week after surgery, but declined thereafter (Table 2).

**Figure 5.**
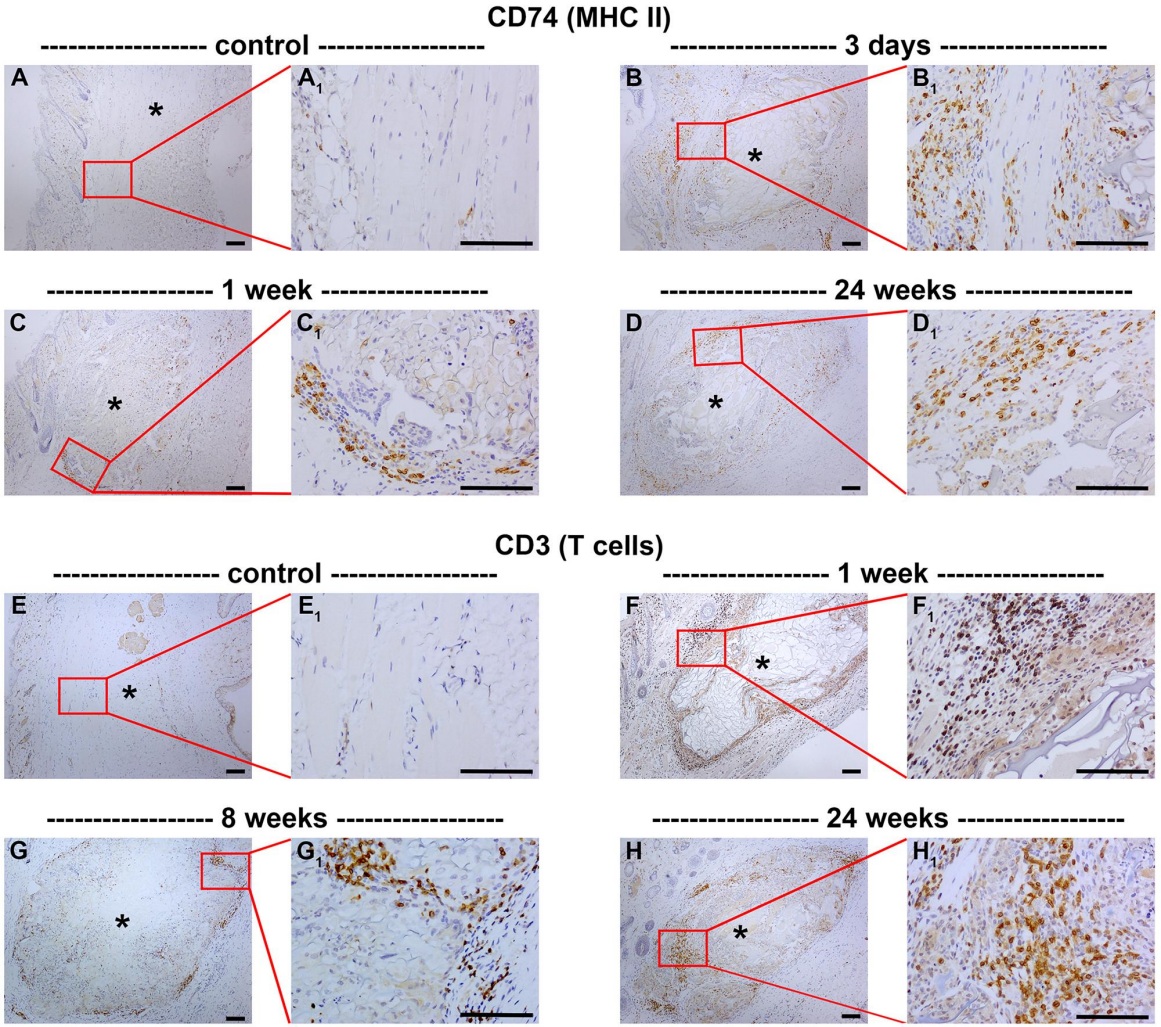
Representative images of antigen-presenting cells and T cells in paraffin-embedded sections of rat eyelids implanted with chitosan scaffolds, as delineated by colorimetric immunohistochemistry for CD74 (**A–D**) and CD3 (**E–H**), respectively. At each time point, low (**A–H**) and corresponding high (**A_1_–H_1_**) magnification images are shown. (**A**) In normal tissue, CD74^+^ cells are scattered throughout the eyelid, but are concentrated in the epidermis. (**B**) At 3 days following surgery, CD74^+^ cells were prevalent throughout the eyelid. (**C**) By 1 week after surgery, CD74^+^ cells were clustered around the scaffold. (**D**) By 24 weeks after surgery, CD74^+^ cells were concentrated both within and surrounding the scaffold. (**E**) In normal eyelid tissue, CD3^+^ cells are not observed. At 1 (**F**), 8 (**G**) and 24 (**H**) weeks following surgery, CD3^+^ cells were observed at the scaffold-tissue interface. Small clusters of CD3^+^ cells were observed within the scaffold, particularly at the longest time point after surgery. Asterisks represent scaffolds. Scale bars: 100 µm.

Next, we explored the involvement of the acquired immune system after implantation of a chitosan scaffold. Presentation of MHC class II molecules to T cells, consequent to processing of foreign proteins, is the major function of antigen-presenting cells—including dendritic cells and macrophages—and the bridge between the innate and acquired immune systems. Thus, we immunolabelled tissue sections with an antibody (CD74) that binds to the invariant chain of MHC class II. In normal tissue, CD74^+^ cells were observed only in the epidermis (Figure 5A). At 3 days following surgery, CD74^+^ cells were prevalent throughout the eyelid (Figure 5B). Thereafter, CD74^+^ cells were clustered around, and in the outer portion of, the scaffold (Figure 5C and D, Table 2). In sham-operated rats, there was an increase in CD74^+^ cells for the first week after surgery (Table 2).

To delineate the presence of T cells, we employed an antibody directed against CD3, a pan T-cell marker [36]. In normal eyelid tissue, CD3^+^ cells were not observed (Figure 5E). At each time point after surgery, clusters of CD3^+^ cells were observed at the scaffold-tissue, with occasional CD3^+^ cells within the scaffold itself (Figure 5F–H, Table 2). In sham-operated rats, there was an increase in CD3^+^ cells for the first week after surgery (Table 2).

### Foreign body reaction, scaffold degradation and integration

**Figure 6.**
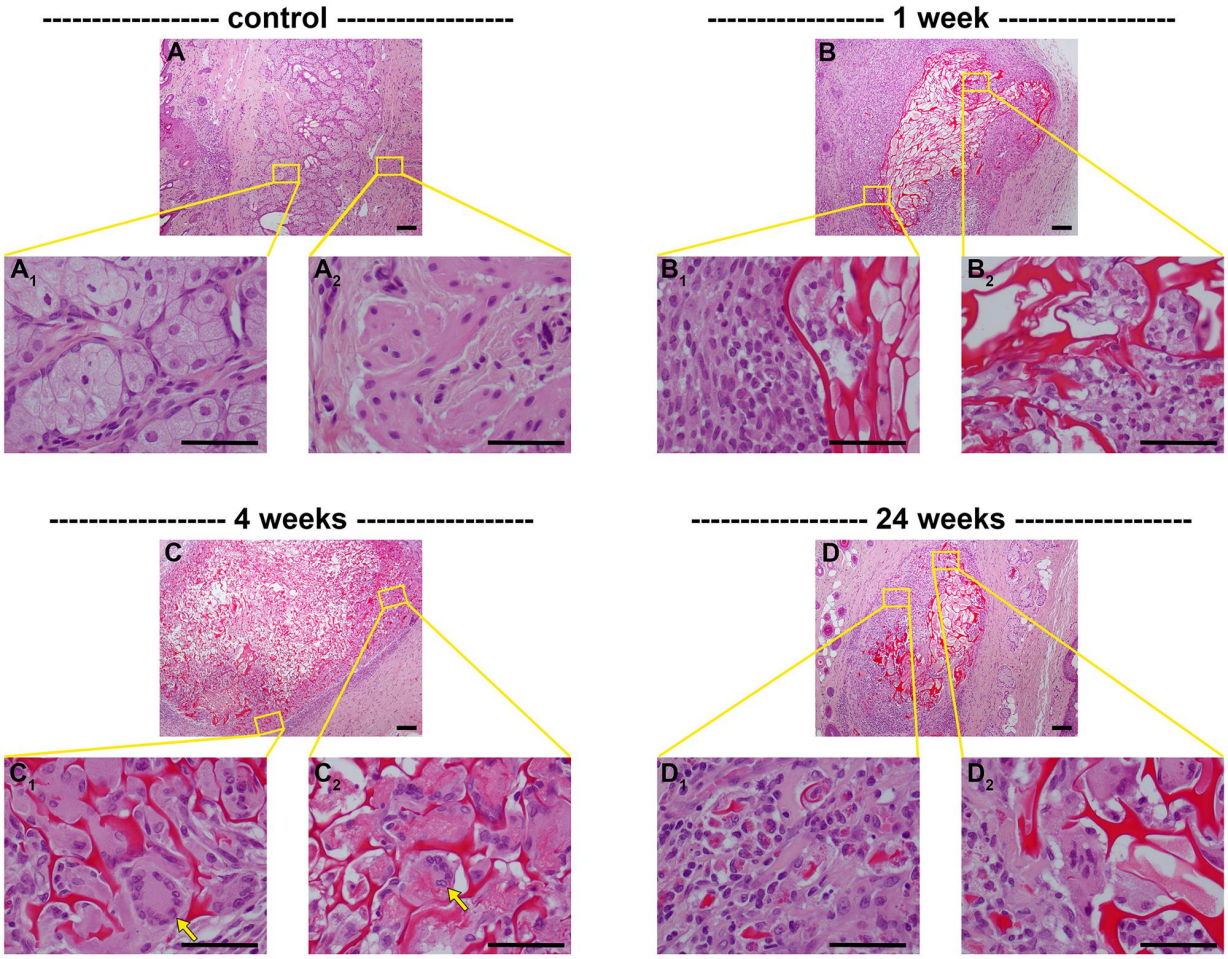
Representative H&E images of implanted chitosan scaffolds in paraffin-embedded sections of rat eyelids. At each time point, low (**A–D**) and corresponding high (**A_1_, A_2_–D_1_, D_2_**) magnification images are shown. (**A**) In normal eyelid tissue, inflammatory cells are not readily apparent. (**B**) At 1 week following implant surgery, chitosan scaffolds remained intact, but inflammatory cells had surrounded and begun infiltrating the scaffold. (**C**) At 4 weeks following surgery, scaffolds had begun to fragment. Fibroblasts, macrophages, and multinucleated foreign body giant cells (arrows) were evident within the periphery of the scaffold. (**D**) By 24 weeks following surgery, scaffolds had undergone substantial fragmentation and degradation. Inflammatory cells and fibroblasts were observed throughout the scaffold. Phagocytosed (engulfed) chitosan was present in the cytoplasm as particulate material or larger fragments of varying size. Asterisks represent scaffolds. Scale bars: overview = 100 µm; **A–D** insets = 50 µm; **E–I** insets = 100 µm.

In the tissue response to biomaterials, chronic inflammation usually leads to the foreign body reaction, characterized by the formation of foreign body giant cells and fibrous encapsulation of the implant [30]. Foreign body giant cells are very large, multinucleated, phagocytic cells formed by the fusion of many macrophages that are, individually, unable to internalize and phagocytose the biomaterial. The presence of foreign body giant cells was discerned by analysis of H&E-stained tissue sections (Figure 6). Foreign body giant cells were not apparent within the first week after implant surgery but were readily observed within the periphery of the scaffold from 4 weeks onwards (Figure 6C and D; arrows). At 24 weeks, most scaffolds had undergone moderate to substantial degradation and engulfed chitosan was present in the cytoplasm of infiltrated cells as particulate material or larger fragments (Figure 6D).

**Figure 7.**
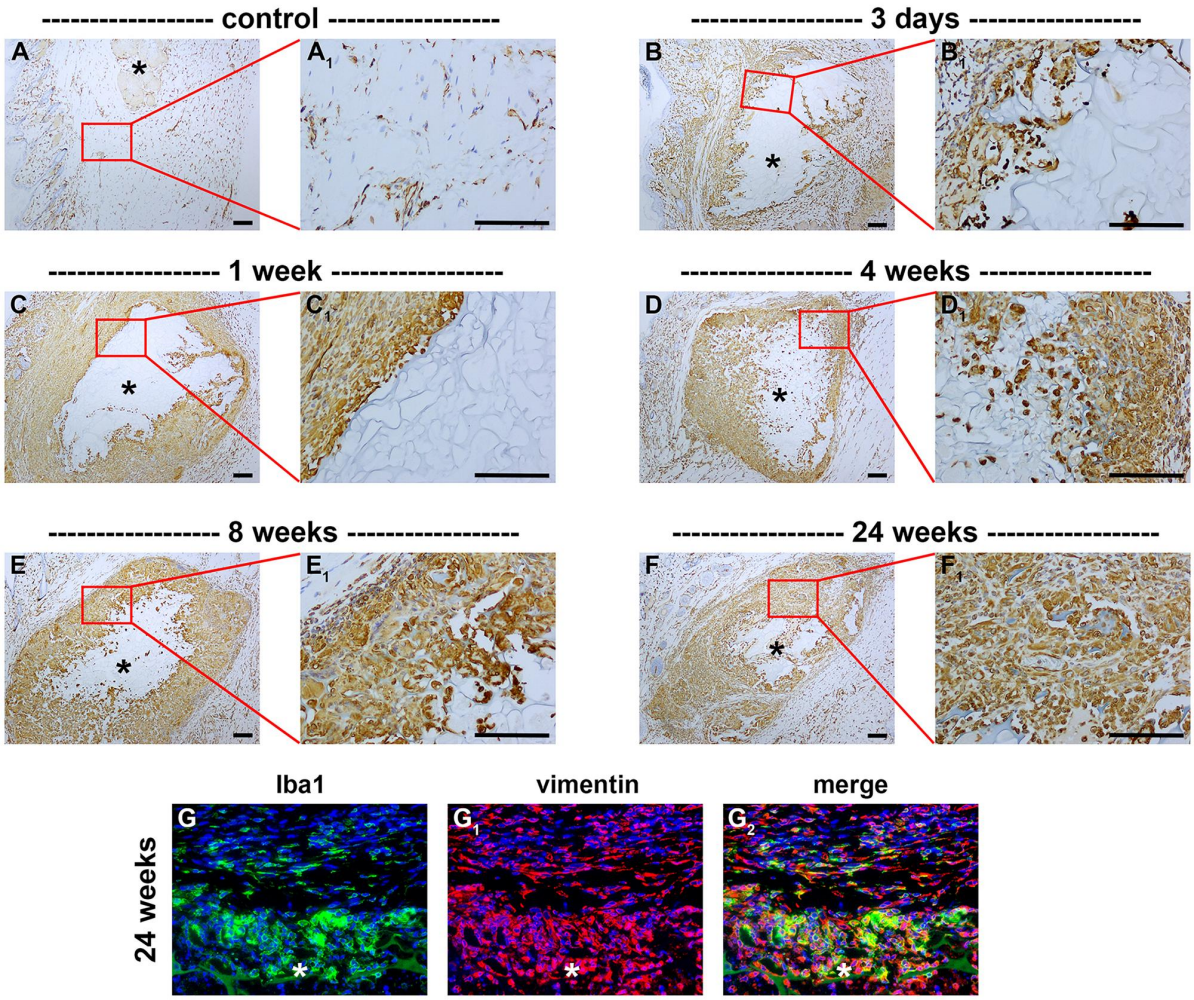
Representative images of mesenchymal cells in paraffin-embedded sections of rat eyelids implanted with chitosan scaffolds, as delineated by colorimetric immunohistochemistry for vimentin. At each time point, low (**A–F**) and corresponding high (**A_1_–F_1_**) magnification images are shown. (**A**) Vimentin^+^ cells are scattered throughout the normal eyelid. (**B**) At 3 days following implant surgery, there was a marked increase in vimentin^+^ cell density throughout the eyelid. (**C**) By 1 week after surgery, vimentin^+^ cells were concentrated in the peri-scaffold region. (**D–F**) At later time points vimentin^+^ cells were abundant within the scaffold and at the scaffold-tissue interface. (**G**) Double labelling of vimentin with the macrophage/dendritic cell marker Iba1 reveals that vimentin co-localized with Iba1 in many immune cells (vimentin^+^-Iba1^+^ cells) both within and outside the implant. Vimentin^+^-Iba1− cells, likely fibroblasts, were similarly present both within and outside the implant. Asterisks represent scaffolds. Scale bars: 100 µm.

Infiltration and proliferation of fibroblasts occurs in response to factors secreted by macrophages and foreign body giant cells. Analysis of H&E-stained tissue sections revealed that cells with the morphological appearance of fibroblasts were present at the tissue-implant interface in the first week post-surgery and thereafter gradually infiltrated the scaffold (Figure 7). To confirm this, immunolabelling of tissue sections for vimentin, which labels mesenchymal cells including fibroblasts, showed an increase in mesenchymal cell density throughout the eyelid by 3 days after surgery (Figure 7B). By 1 week, mesenchymal cells were concentrated at the tissue-scaffold interface (Figure 7C, Table 2). At later time points, mesenchymal cells were abundant both within the scaffold and at the interface (Figure 7D–F, Table 2). Double labelling of vimentin with the monocyte/macrophage/dendritic cell marker Iba1 revealed the presence of fibroblasts (vimentin^+^-Iba1^-^ cells) both inside and outside the scaffold (Figure 7G). In sham-operated rats, there was an increase in mesenchymal cells for the first week after surgery (Table 2).

**Figure 8.**
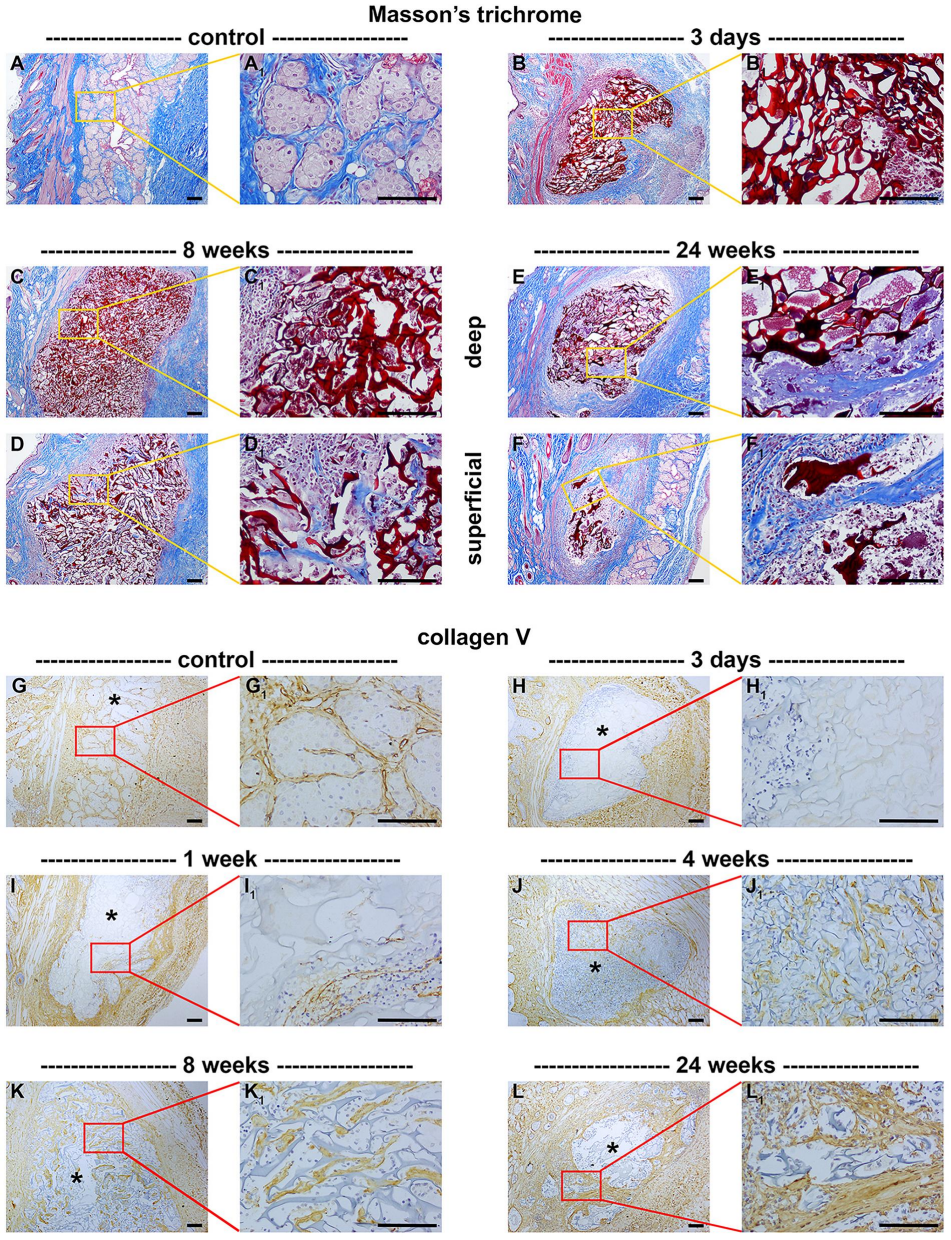
Representative images of extracellular matrix (ECM) deposition in paraffin-embedded sections of rat eyelids implanted with chitosan scaffolds, as delineated by Masson’s trichrome staining (**A–F**) and colorimetric immunohistochemistry for collagen V (**G–L**). At each time point, low (**A–L**) and corresponding high (**A_1_–L_1_**) magnification images are shown. (**A**) The tarsal plate of the normal eyelid features extensive ECM tissue (blue). The high magnification image shows connective tissue stroma surrounding meibomian glands. (**B**) At 3 days following implant surgery, no ECM was present within the scaffold. (**C, D**) By 8 weeks after surgery, ECM had largely encapsulated the scaffold and ECM strands were observed within the more superficial layers of the fragmenting scaffold (**D**), but not within the core (**C**). (**E, F**) by 24 weeks after surgery, there was extensive deposition of ECM within the degenerating scaffold, particularly within the more superficial layers (**F**). (**G**) Collagen V is abundant in the normal eyelid. (**H–J**) Over the 24-week period studied following implant surgery, there was a progressive increase in collagen V deposition throughout the scaffold. Asterisks represent scaffolds. Scale bars: 100 µm.

**Figure 9.**
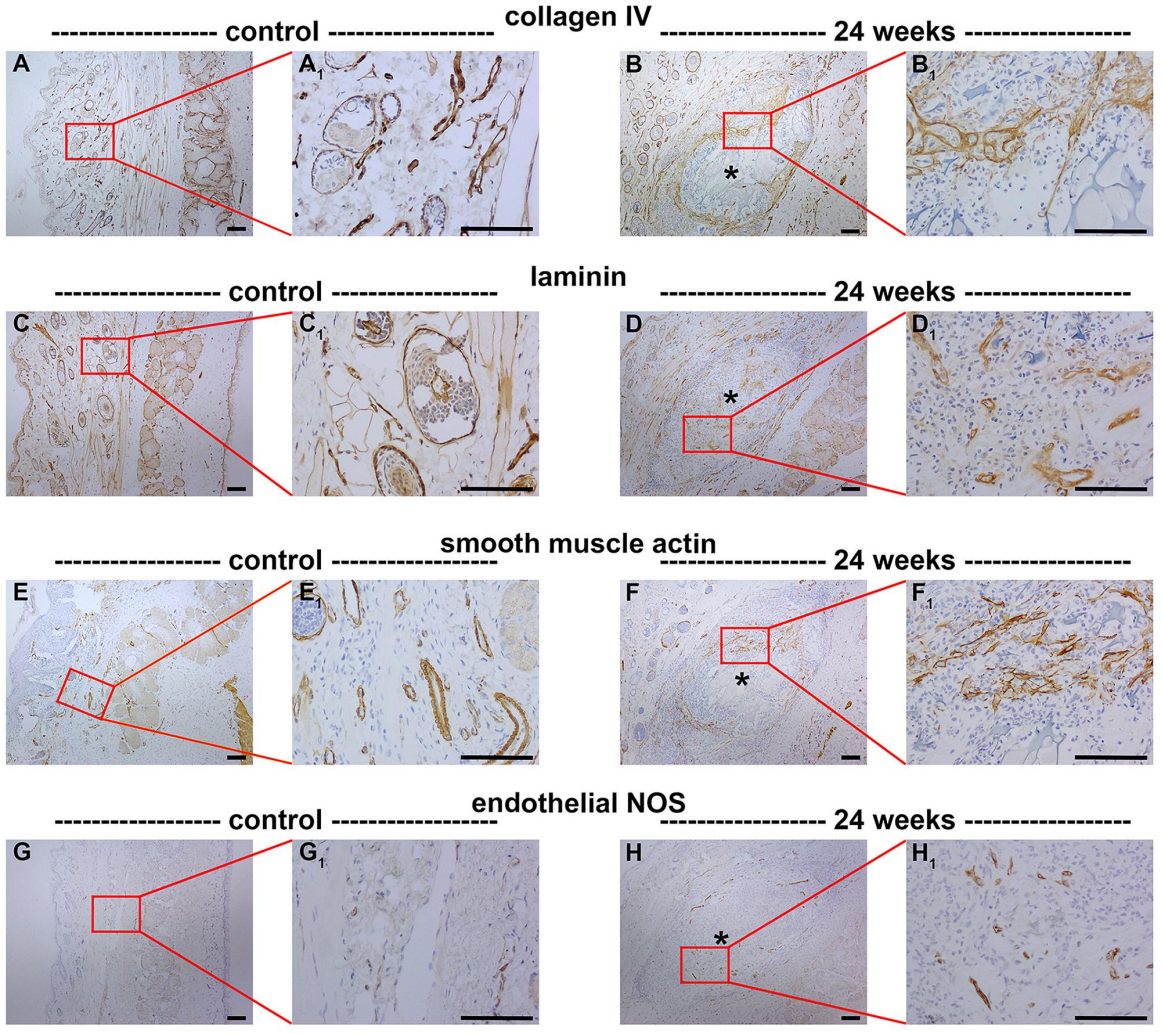
Representative images of collagen IV, laminin, α- smooth muscle actin (αSMA) and endothelial nitric oxide synthase (NOS) deposition in paraffin-embedded sections of rat eyelids implanted with chitosan scaffolds, as delineated by colorimetric immunohistochemistry. Low (**A–H**) and corresponding high (**A_1_–H_1_**) magnification images are shown. (**A, C**) In the normal eyelid, collagen IV and laminin localize to the basement membrane zone. (**B, D**) At 24 weeks following implant surgery, a network of collagen IV and laminin was observed in the scaffold. (**E**) In the normal eyelid, αSMA localizes to differentiated smooth muscle cells with contractile capacity, including vascular smooth muscle cells. (**F**) At 24 weeks following implant surgery, a network of αSMA was observed in the scaffold. (**G**) In the normal eyelid, endothelial NOS localizes to vascular endothelium. At 24 weeks following implant surgery, scattered fragments of endothelial NOS labelling was observed within the scaffold. Asterisks represent scaffolds. Scale bars: 100 µm.

In most cases of biomaterial implantation, a fibrous capsule of varying thickness and vascularity forms around the implant, isolating the implant from the host tissue. For tarsal plate repair, the preferred outcome is slow degradation of the implanted scaffold with integration into the tissue, culminating in regeneration of a structural and functional tarsus. As shown by Masson’s trichrome staining (Figure 8A–F) as well as immunolabelling for collagen V (Figure 8G–L) and collagen VI (data not shown), a thin fibro-collagenous capsule surrounded the chitosan scaffold during the weeks after eyelid implantation. As previously described, this encapsulation did not prevent integration of mesenchymal cells into the scaffold, which effected a slow degradation of the scaffold over the 24-week evaluation period post-surgery (Table 2); after 24 weeks we observed a 72.5 ± 15.3% degradation of the scaffold (*n* = 6 eyelids). At each time point, chitosan degradation was more advanced in the superficial portion of the implant and less advanced in the deeper portion of the implant (Figure 8). This degradation occurred concurrently with deposition of various ECM components within the scaffold including collagens V and VI (Figure 8, Table 2), the major basement membrane constituents collagen IV and laminin (Figure 9A–D, Table 2), and α-smooth muscle actin (αSMA; Figure 9E–F), which normally localizes to differentiated smooth muscle cells with contractile capacity, including vascular smooth muscle cells. Finally, there were fragments of blood vessels within the scaffold, as evidenced by immunolabelling for endothelial nitric oxide synthase (Figure 9G–H).

## Discussion

The eyelid tarsus represents an excellent candidate for bioengineering, being a thin discrete structure with well-characterized histological and biomechanical properties which is easily surgically accessible [37]. The biopolymer chitosan ostensibly fulfils the necessary criteria for a tarsal substitute, comprising a versatile porous structure with non-toxic degradation products that is amenable to cellular infiltration [38, 39]. To improve biocompatibility and address the inherent distribution of both hydrophobic and hydrophilic side-groups within chitosan [38, 39], we have produced a hydrogel preparation of this polymer, not only improving the tissue compatibility, but also incorporating the advantages of hydrogels, namely, suitable strength, stability and moldability [32]. Furthermore, the mechanical properties of our chitosan hydrogels: 0.17 ± 0.01 MPa initial modulus, 0.62 ± 0.1 MPa final modulus and 22.1 ± 0.02% elongation, were closely comparable to those of human tarsus tissue: 0.14 ± 0.1 MPa initial modulus, 1.7 ± 0.6 MPa final modulus and 16.0 ± 2.0% elongation, identifying them as suitable candidates for tarsal substitution [32]. In the present study, therefore, we aimed to further investigate the suitability of utilizing our chitosan hydrogels as tarsal surrogates by implanting them into rat eyelids and evaluating their inflammatory response and integration into native tissue over time.

Implantation of a biomaterial into a living tissue induces a complex cascade of events known as the FBR. The FBR is generally considered to encompass several overlapping phases: blood–biomaterial interactions, acute inflammation, chronic inflammation, foreign body giant cell formation, and fibrous capsule formation [29]. Curiously, even inert, non-toxic biomaterials instigate the FBR [40]. Originally, the FBR was considered an adverse reaction that needed to be minimized. Biomaterial implants were fabricated not only to encompass desirable physical and mechanical properties, but also to be immunologically passive, in order to prevent the instigation of a significant host response and to ensure a successful clinical outcome [41]. It is now believed, however, that both scaffold integration and performance will likely benefit from the utilization of biomaterials that produce a controlled immunogenicity, since this action itself creates a conducive bioactive environment with improved tissue healing and regeneration [42]. Thus, the modest immune reaction elicited by implanted chitosan is a desirable tissue response [23].

During the acute inflammation phase, circulating neutrophils and mast cells infiltrate the tissue surrounding the implant in response to blood–biomaterial interactions or tissue damage caused by surgery. These cells phagocytose debris, degranulate, and release chemoattractants which attract monocytes and lymphocytes to the implant site, thereby establishing and influencing the chronic inflammatory phase of the process. Neutrophils are typically only resident for a few days after scaffold implantation; however, they have been shown to persist for several weeks around implanted biomaterials [43], including after introduction of unsaturated polyester scaffolds in rabbit tarsus [15]. There is evidence to suggest that a disproportionate neutrophil response promotes fibrous encapsulation, hindering tissue–biomaterial integration [41]. In the current study, neutrophils were abundant at the implant-tissue interface at 3 days following implant surgery, but their prevalence rapidly declined thereafter, and they were barely detectable at time points later than 1 week post-surgery. This is consistent with earlier studies that have shown chitosan to stimulate neutrophil infiltration and migration [44]. Furthermore, this agrees with a previous study where PHBHHx scaffolds were implanted into rat eyelids and a high density of neutrophils infiltrated into the implanted region within the two weeks following surgery but markedly declined thereafter [18]. Conversely, however, the same study demonstrated that transplanted acellular dermal matrices caused no inflammatory response up to 8 weeks post-surgery [18]. Mast cells are specialized granulocytic leukocytes that have a critical function in wound healing and tissue repair in response to foreign bodies, including synthetic and naturally derived biomaterials [45, 46]. Previous *in vivo* and *in vitro* studies have shown that chitosan biomaterials can activate mast cells [45, 47]. We did not find an increased presence of mast cells in response to chitosan scaffold implantation; however, it must be recognized that the earliest time point analysed was 3 days post-surgery and the acute inflammatory stage of the FBR is short-lived. It is plausible that a mast cell response occurred prior to the first point of analysis.

Monocyte-derived macrophages play the major role in the FBR to implanted biomaterials, regulating both the duration and outcome of wound healing [30]. They represent a heterogeneous population of cells with differing polarization states and functions. Macrophages are routinely classified into ‘classically-activated’ M1-type macrophages or ‘alternatively-activated’, M2-type macrophages. This classification is oversimplistic, however, as multiple subtypes of M2 macrophages have been identified. Moreover, macrophages at the site of implanted biomaterials have been shown to display cellular plasticity, fluctuating between these two distinct phenotypic states [48]. M1 macrophages, which express a high level of iNOS, are pro-inflammatory, highly phagocytic, and effective antigen-presenting cells via cell surface expression of MHC II. They endeavour to break down biomaterials via phagocytosis, and via secretion of pro-inflammatory cytokines, lysosomal enzymes and reactive oxygen and nitrogen species. We observed iNOS^+^ M1 macrophages surrounding the implant within the first week after surgery, constituting part of the acute inflammatory stage of the FBR and initiating phagocytosis of foreign material. Thereafter, iNOS^+^ macrophages were concentrated within the scaffold. This is predictable because chitosan, as an implant, is designed to be degraded and phagocytosed, a function that is normally associated with M1 macrophages. The M2 phenotype of macrophage is anti-inflammatory, pro-regenerative and is associated with both the chronic inflammatory stage of the FBR and with wound healing via tissue remodelling, although a disproportionate quantity of M2 macrophages may result in fibrotic encapsulation of the implant [49]. We observed CD206^+^ M2 macrophages at the scaffold-tissue interface at all time points after surgery, but relatively few of these cells were present within the scaffold itself. These results suggest that M2 macrophage-directed ECM remodelling was most active at the periphery of the scaffold. Implantation of PHBHHx scaffolds into rat eyelids also gave rise to an increase in macrophages in the acute inflammatory phase, but these declined by 4–8 weeks post-surgery, likely indicating that these were M1 class cells [18].

Interestingly, macrophage polarization following implantation of porous chitosan scaffolds appears to be related to the degree of acetylation. Chitosan scaffolds with a low degree of acetylation (2–5%) induce primarily an M2 phenotype in adherent and invading macrophages, whereas scaffolds with a higher degree of acetylation (15%) induce a greater M1 response [50, 51]. The scaffolds employed in this study have a higher degree of acetylation (17.5–22.4%), which may explain the predominance of M1 macrophages. It would be interesting in future work to test chitosan scaffolds with an even greater level of deacetylation, although this would also alter its rate of degradation.

During the FBR to implanted biomaterials, a proportion of macrophages fuse to become foreign body giant cells. This process occurs as a result of the failure of individual macrophages to engulf and phagocytose large fragments of biomaterial [52]. In certain situations, foreign body giant cells are considered an undesirable presence, being strongly linked to ongoing inflammation, unwanted fibrous encapsulation and implant deterioration [30]; however, for temporary, biodegradable implants, tissue regeneration and healing is dependent upon an orderly degradation of the biomaterial. The number of foreign body giant cells has been found to correspond to the speed of biomaterial degradation [52]. Accordingly, for tarsal plate replacement implants, the presence of foreign body giant cells likely contributes to the tissue regeneration process. We have shown, here, that foreign body giant cells are observed after 1 week post-surgery and remain for at least the first 8 weeks. This is in complete agreement with addition of PHBHHx scaffolds into rat eyelids [18], and poly(propylene fumarate)-*co*-2-hydroxyethyl methacrylate (PPF-HEMA) into rabbit eyelids [15], where macrophages and giant cells were documented at 1–8 weeks post-implantation.

During the chronic inflammatory phase of the FBR, infiltration of lymphocytes—principally CD4^+^ T cells—to biomaterial implant sites is routinely observed, but their roles remain poorly understood. T cells are believed to have an important influence on the balance between inflammation, tissue regeneration and fibrosis [53], and have been proposed to guide the switching of macrophages from an M1 to M2 phenotype. In the current study, T cells were observed at the scaffold-tissue interface at all times following surgery, forming part of the acute and chronic inflammatory stage of the FBR. They were scarcer than other immune cells, suggesting a modulatory role on tissue homeostasis. We also observed CD74^+^ MHC II cells, likely dendritic cells and/or M1 macrophages, throughout the eyelid in the acute inflammatory phase and thereafter, in the chronic phase surrounding scaffold material, playing a role in recruitment of other immune cells.

As previously described, the porous chitosan scaffold permitted rapid infiltration of immune cells—largely macrophages—and the fusion of a subset of these macrophages into foreign body giant cells. This resulted in a gradual phagocytosis of the scaffold over the time course of the study, which remained, however, incomplete by 24 weeks post-surgery. For successful wound healing, the biodegradable scaffold must be organically replaced by connective tissue, thereby reproducing the strength and stability of the tarsal plate [20]. ECM synthesis and remodelling is accomplished by fibroblasts, which migrate to implant sites and proliferate in response to signals from macrophages. For nearly all biomaterial implants, fibroblast activity leads to the formation of a fibrous capsule around the implant, which can vary in thickness and vascularity. For a biodegradable implant, the capsule should not be too thick or long-lasting, as this causes the implant to be isolated from the host, impacting nutrient and product diffusion and hindering bio-integration and tissue regeneration [54]. ECM remodelling within the scaffold can be achieved by fibroblasts located within the scaffold itself, or, for porous biocompatible materials such as chitosan, by fibroblasts located at the scaffold-tissue interface, since ECM can penetrate the scaffold via the pore network. We used Masson’s Trichrome histological method to stain all connective tissue, and immunohistochemical labelling to examine some individual components of the ECM. Our data revealed that the chitosan scaffold offers great promise as a tarsal substitute. A fibrous capsule formed around the implant within the first week after surgery, which by 4 weeks was very thin. At this point, fibroblasts and collagen fragments were scattered throughout the scaffold. Over the following weeks, deposition of collagen within the scaffold became more extensive and inter-connected, concurrent with scaffold degradation. This agrees with previous work detailing deposition of collagen within chitosan pores [55] and within regenerated tarsal tissue after implantation of poly(lactide-*co*-glycolide) (PLGA) scaffolds pre-seeded with bone marrow stem cells and a TGF-β1 construct [13]. In the present study, the process was only partially complete at 24 weeks and varied between samples but was progressing in a predictable manner. In addition to the deposition of collagenous ECM, a partial network of basement membrane constituents and fragments of blood vessels were also evident at the 24-week time point. Adequate neovascularization of the scaffold is an eventual requirement to ensure survival and functionality of the regenerated tissue, but unlike with surgical implantation of grafts [20] or tissue-engineered constructs [56], there is no immediate requirement for a vascular supply when using an acellular approach.

The present study employed two control groups: untreated rats and sham-operated rats. This approach permits differentiation between chitosan implant-specific tissue responses versus those caused purely by the surgical procedure. The results showed that there was a transient increase in immune cells, predominately macrophages, immature dendritic cells and T cells, following sham surgery. This is to be expected given the physical injury that ensues after eyelid incision. No longer-term immune reaction was observed. Two conclusions can be drawn: firstly, immune responses surrounding the chitosan scaffold at time-points beyond 1 week post-surgery are prompted by the biomaterial implant rather than by the surgery, and secondly, that within the first week post-surgery, inflammatory responses within the eyelid reflect a combination of physical trauma as well as a chitosan-induced bioreaction.

## Conclusion

The ideal sequence of regenerative local tissue events after implantation of an engineered scaffold material would include: (i) surgical recovery, (ii) acute and then chronic immune responses, (iii) establishment of the correct intercellular milieu including ECM deposition, (iv) infiltration and incorporation of native cells by de-differentiation, migration and proliferation, (v) vascularization of the regenerating zone and (vi) re-differentiation of incorporated cells, resulting in requisite nervous innervation and re-establishment of true tissue function. These events should occur alongside, and subsequent to, gradual degradation and phagocytic removal of the scaffold material. Herein, we have assessed the outcome of implantation of chitosan hydrogel scaffolds into rat eyelids for 24 weeks. By this time point we observed extensive tissue remodelling occurring, including degradation of the scaffold, ECM deposition, cellularization of the implanted region, as well as rudimentary vascular growth. As such, although the present study provides evidence to support the use of chitosan hydrogel in tarsus remodelling, a major limitation is that the time course was not extended to the point of full scaffold degradation. Thus, future work is needed to assess longer-term tissue events, ideally detailing complete degradation of the implant and full regeneration of the tarsus. This would particularly be the case should the scaffold be tested in human patients. The significance of the present study, however, is the description of biomaterial implantation into eyelid tissue, in terms of detailed temporal individual cell type responses. Additionally, the biomechanical properties of the chitosan hydrogel scaffolds used in this study have been previously shown to mimic the native human tarsus, including in stress–strain characteristics [32].

The use of rats, per se, is an additional limitation of this study. Although rat eyelids have a similar basic morphology to that of a human, they are obviously much smaller. Thus, application of a chitosan hydrogel implant of the same dimensions to a human eyelid would take up relatively less tissue volume, potentially engendering a different response. Nonetheless, the rat was deemed a practical initial animal model for investigating the use of chitosan hydrogels in tarsal repair. Any additional studies, however, would be more pertinently carried out on an alternative animal species, such as a rabbit. Not only does the rabbit also have a structurally similar eyelid to a human, but their eyelid is obviously much larger. Furthermore, rabbits would be more practical to utilize than rats for a longer-term assessment, owing to their longer healthy lifespan.

A further experimental aim will be to determine whether complete tissue regeneration can be obtained by implanting cellularized chitosan scaffolds. *In vitro* incorporation of cells, such as patient-derived stem cells, propagated from the individual receiving the implant represents the concept of ‘personalized’ treatment [57]. Synthetic 3D-printed poly-caprolactone matrices seeded with secretory sebocytes, for example, have been shown to integrate well after transplantation into nude mice [16], and additionally, 3D-bioprinted matrices of atelocollagen/alginate incorporating human adipose-derived stem cells (hADSCs), not only demonstrated good integration but also expressed lipid-secreting cells resembling meibocytes after implantation [17]. We have already proven that our chitosan hydrogel scaffolds are amenable to incorporation of ocular fibroblasts [32] and it thus remains to assess the outcomes of surgical implantation of these cellularized matrices into the eyelid.
